# Impact of a Standardized Gastrografin Protocol on Small Bowel Obstruction Outcomes at a Community Hospital: A Retrospective and Prospective Cohort Study

**DOI:** 10.7759/cureus.90761

**Published:** 2025-08-22

**Authors:** Ryan Korlewitz, Mark Glover, Fariha Tareen, Meghan Linz, Amy Braddock

**Affiliations:** 1 General Surgery, Trinity Health Oakland/Wayne State University, Pontiac, USA; 2 Clinical Foundations, Ross University School of Medicine, Bridgetown, BRB

**Keywords:** adhesions, adhesive disease, community hospital, gastrografin, hyperosmolar, nonoperative intervention, oral contrast, protocol, small bowel, small bowel obstruction

## Abstract

Small bowel obstruction (SBO) is a common condition in general surgical practice. This study aimed to determine whether the use of an oral hyperosmolar contrast agent (Gastrografin) combined with serial abdominal radiographs improves patient outcomes compared with standard management of SBO in a community hospital. A retrospective and prospective analysis was conducted of patients admitted with SBO over a two-year period. The retrospective control group included patients from January 2022 through December 2022, while the prospective group included those from January 2023 through December 2023. Exclusion criteria were emergent operative intervention, recent abdominal surgery (<30 days), and age <18 years. Patients treated with a standardized Gastrografin protocol were compared with those managed conventionally. Primary outcomes included length of stay (LOS), number of CT scans, progression to surgery, and 30-day readmission rates. Ninety patients met the inclusion criteria; 24 received the Gastrografin protocol, and 66 received standard care. The Gastrografin group had a significantly shorter LOS (2.2 ± 1.4 vs. 4.6 ± 4.2 days, p < 0.001) and underwent fewer CT scans (1.0 ± 0.0 vs. 1.4 ± 0.6, p < 0.001). Rates of surgical intervention (4.2% vs. 21.2%, p = 0.06) and 30-day readmission (12.5% vs. 15.2%, p = 1.00) did not differ significantly between groups. The findings suggest that a standardized Gastrografin protocol may reduce hospital LOS and CT utilization in the management of SBO at community hospitals.

## Introduction

Adhesions from prior abdominal surgery are the leading cause of small bowel obstruction (SBO) in developed countries, followed by hernias and other causes such as malignancy, inflammatory bowel disease, volvulus, and retained foreign bodies [1]. In the United States, adhesive SBO accounts for approximately 15-20% of all surgical admissions, resulting in more than 300,000 operations and over 850,000 cumulative hospital days annually [2-4]. The reported incidence after major abdominal surgery is as high as 30%, and SBO contributes to approximately 20% of surgical emergencies [5,6].

Traditional management includes bowel rest, nasogastric (NG) decompression, IV fluid resuscitation, and serial abdominal examinations. However, distinguishing between patients who will respond to conservative management and those who may require operative intervention remains a significant challenge. This diagnostic uncertainty can lead to repeated imaging, prolonged hospitalization, and increased ionizing radiation exposure from CT scans [5,6].

Gastrografin^®^ (Schering AG, Berlin, Germany), a hyperosmolar, water-soluble contrast agent composed of sodium diatrizoate and meglumine amidotrizoate, is commonly used for GI imaging [7]. This contrast medium enhances visualization of the GI tract compared with standard noncontrast radiographs. Gastrografin has an osmolarity of approximately 1900 mOsm/L, about six times greater than that of extracellular fluid [7]. Its hyperosmolarity draws water into the bowel lumen via osmotic gradients, thereby reducing intestinal wall edema and stimulating peristalsis [8]. This may help relieve obstruction by increasing the pressure gradient across the obstruction and promoting fluid shifts from the intramural to intraluminal space [8]. Thus, Gastrografin is hypothesized to have both diagnostic and therapeutic effects in patients with suspected adhesive SBO.

Previous studies from academic centers have demonstrated the beneficial effects of Gastrografin in adhesive SBO. Cohen et al. reported that administration of an oral hyperosmotic contrast agent (e.g., Gastrografin) within 12 hours of presentation for adhesive SBO was associated with a shorter hospital stay, fewer complications during admission, and a lower one-year mortality rate [9]. In a study of 161 patients with adhesive SBO, Chen et al. found that 98% of patients responded to conservative therapy when oral contrast was observed in the colon within 24 hours of administration [10]. Although adhesive SBO is encountered in nearly every hospital in the developed world, management practices vary significantly [11].

Despite strong evidence from academic centers, literature on the use of standardized Gastrografin protocols in community hospitals remains limited [11]. Community hospitals manage a large proportion of SBO cases, yet standardized protocols are underutilized [11,12]. We hypothesized that the development and implementation of a standardized oral hyperosmolar contrast challenge for adhesive SBO would reduce hospital length of stay (LOS), decrease the number of CT scans performed during admission, lower the need for surgery, and reduce 30-day readmissions for SBO at our community hospital.

This article was previously presented as a meeting abstract at the 2024 Michigan Summit for Quality Improvement and Patient Safety on May 22, 2024.

## Materials and methods

Study design

This combined retrospective and prospective study was conducted at a single community hospital after approval from the Trinity Health Oakland Institutional Review Board (approval 2023-011). Retrospective data were collected from January 2022 to December 2022 for the control group using ICD-10 code K56.609 for SBO. The diagnosis of SBO was confirmed by a chart review of CT reports. A total of 100 patient charts were initially screened in the control group.

Exclusion criteria included abdominal surgery within 30 days of presentation, pediatric patients (<18 years), nonadhesive SBO (e.g., tumor and Crohn’s disease), and patients who underwent emergency surgery upon evaluation. Emergency surgery was defined as surgery performed within six hours of surgical consultation due to hemodynamic instability, physical examination findings, or CT findings.

**Figure 1:**
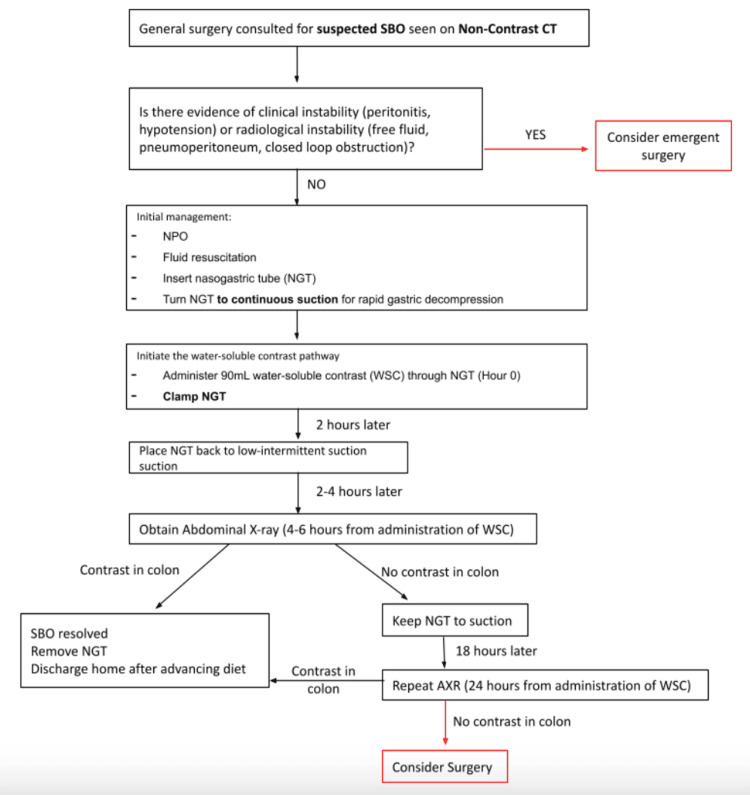
Gastrografin protocol developed and administered to study patients AXR, abdominal radiograph; NGT, nasogastric tube; NPO, nil per os; SBO, small bowel obstruction; WSC, water-soluble contrast Image credits: Ryan Korlewitz, Meghan Linz, Kaitlin Zaki-Metias, Stephen Seedial, Mark Glover, and Amy Braddock

A standardized Gastrografin protocol (Figure 1) was jointly developed by the general surgery and radiology departments and implemented in January 2023. Prospective data were collected from patients treated with this protocol upon presentation with SBO between January 2023 and December 2023. Ninety-nine patient charts were initially screened in the protocol group. All patients presenting with SBO during this period were enrolled in the study, as this represented the new standard of treatment on the general surgery service.

Gastrografin challenge protocol

**Figure 2:**
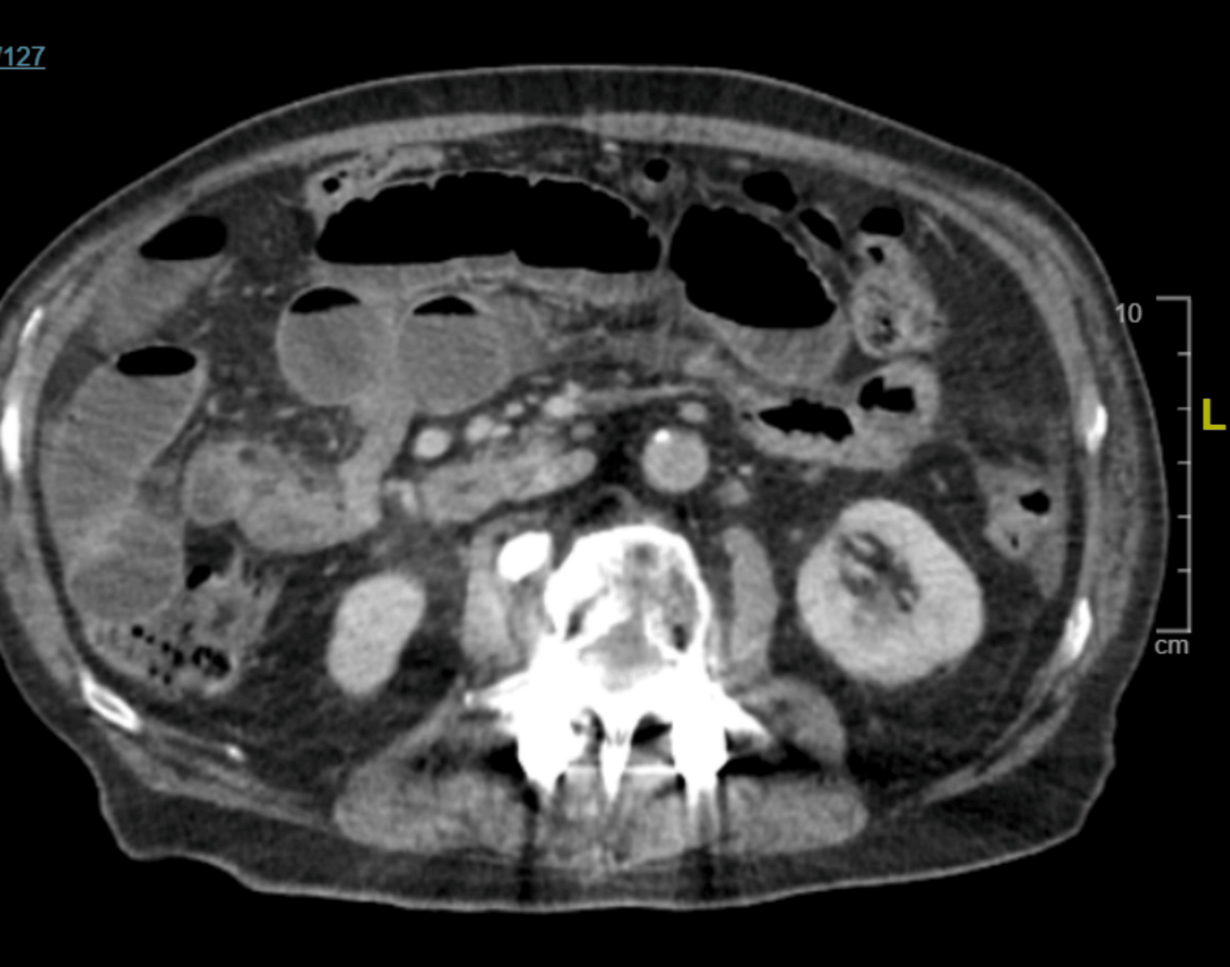
Representative CT image from a patient presenting with SBO SBO, small bowel obstruction

**Figure 3:**
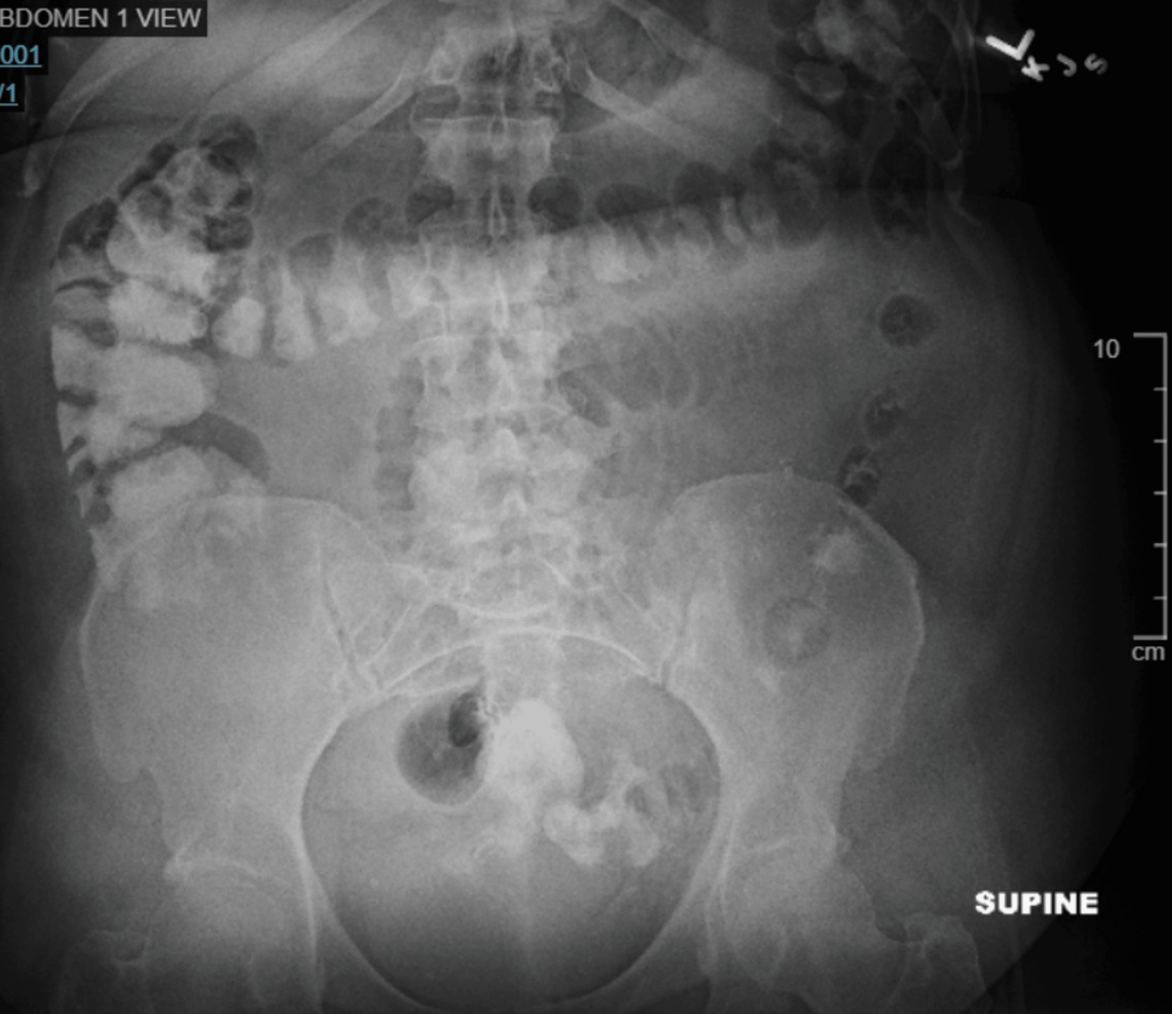
AXR obtained six hours after contrast administration in a patient with SBO AXR, abdominal radiograph; SBO, small bowel obstruction

**Figure 4:**
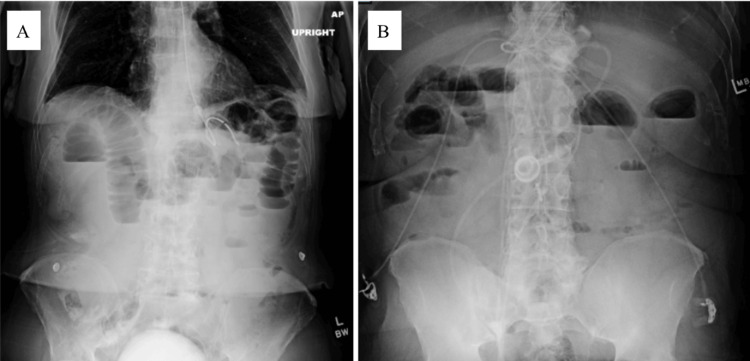
Representative AXRs of control patients (A) AXR demonstrating SBO with dilated loops of small bowel; no oral contrast is visible. (B) AXR demonstrating SBO with prominent air-fluid levels; no oral contrast is visible. AXR, abdominal radiograph; SBO, small bowel obstruction

Patients with a general surgery consultation for suspected SBO on nonoral contrast CT (Figure 2) were first assessed for clinical or radiological instability. Those meeting the study criteria entered the Gastrografin challenge protocol. Patients were made nil per os, received IV fluids, and underwent rapid NG decompression. Gastrografin (90 mL) was then administered via NG tube, which was clamped for two hours. Serial abdominal radiographs (AXRs; Figure 3, Figure 4) were obtained at six and 24 hours post-administration to assess for contrast passage into the colon. If contrast reached the colon, patients’ diets were advanced, and they were discharged when clinically appropriate; if not, surgical intervention was considered.

Data analysis

Numerical data on hospital LOS, number of CT scans, progression to surgery, and 30-day readmission rates were collected through manual chart review. Group comparisons were performed using unpaired t-tests and chi-square tests, as appropriate. Statistical analysis was conducted using IBM SPSS Statistics for Windows, Version 28.0 (Released 2021; IBM Corp., Armonk, NY, USA). All t-tests were two-tailed with an α level of 0.05.

## Results

Study population

A total of 90 patients were included in the final analysis, of whom 24 were managed with the standardized Gastrografin protocol and 66 received conventional management. Baseline demographics, including age, sex, and prior abdominal surgical history, were similar between groups and did not differ significantly, supporting a balanced comparison.

Hospital LOS

Patients managed with the Gastrografin protocol had a significantly shorter average hospital stay compared with the control group. The mean LOS was 2.2 ± 1.4 days in the protocol group versus 4.6 ± 4.2 days in the control group (p < 0.001, Table 1). This 2.4-day reduction represents nearly a 50% decrease in inpatient stay duration, suggesting a clinically meaningful improvement in patient flow and resource utilization.

**Table 1:** Comparison of LOS and number of CT scans between study groups p ≤ 0.05 was considered statistically significant. An asterisk (^*^) indicates a statistically significant difference between groups. LOS, length of stay

Outcome	Gastrografin protocol (n = 24)	Control (n = 66)	t-test value	p-Value
Average LOS (days)	2.2 ± 1.4	4.6 ± 4.2	t(87.665) = -4.004	p < 0.001^*^
Total CT scans during admission	1.0 ± 0.0	1.4 ± 0.6	t(65.000) = -6.195	p < 0.001^*^

The shorter LOS observed in the Gastrografin group may be attributed to earlier resolution of obstruction in patients who demonstrated timely transit of contrast into the colon on serial AXRs, which facilitated expedited clinical decision-making and discharge planning.

CT imaging utilization

There was a statistically significant reduction in the number of CT scans performed during admission in the Gastrografin protocol group. All patients in this group underwent a single noncontrast CT scan at presentation, with no repeat CT imaging required during hospitalization. In contrast, patients in the control group underwent an average of 1.4 ± 0.6 CT scans during admission (Table 1). This finding highlights the effectiveness of the protocol in reducing reliance on repeat imaging, an advantage particularly relevant in community hospitals with limited radiologic resources.

Surgical intervention

Although the difference in operative rates between groups did not reach statistical significance, a notable trend was observed. Only one patient (4.2%) in the Gastrografin group required surgery, compared with 14 patients (21.2%) in the control group (p = 0.06; Table 2). This marked reduction, while not statistically significant, may reflect the therapeutic role of Gastrografin in relieving partial obstructions and supporting nonoperative resolution.

**Table 2:** Comparison of progression to surgery and 30-day readmission between groups p ≤ 0.05 was considered statistically significant.

Outcome	Gastrografin protocol (n = 24)	Control (n = 66)	Chi-square test value	p-Value
Progression to surgery	4.20%	21.20%	X² (1, 90) = 3.682	p = 0.06
30-day readmission rate	12.50%	15.20%	X² (1, 90) = 0.100	p = 1.00

30-day readmission

The 30-day readmission rate for SBO was 12.5% in the Gastrografin protocol group and 15.2% in the control group (p = 1.00; Table 2), indicating no significant difference. These findings suggest that early discharge following clinical resolution of SBO with Gastrografin does not increase the risk of recurrence or complications requiring readmission. Thus, the protocol appears safe from a short-term follow-up perspective.

## Discussion

The implementation of a standardized Gastrografin protocol at our community hospital led to a significant reduction in hospital LOS and CT scan utilization among patients with adhesive SBO. These findings not only support existing literature but also demonstrate the feasibility and benefit of adopting evidence-based SBO management strategies outside tertiary care centers.

Previous studies have reported that the administration of oral water-soluble contrast agents can shorten the duration of SBO and help identify patients who may require surgical intervention. A meta-analysis by Abbas et al. and subsequent reviews have shown that water-soluble contrast challenge tests are associated with higher rates of SBO resolution and a decreased need for surgery [8,9,11]. Furthermore, the early use of oral contrast agents has been shown to shorten hospitalizations and reduce complication rates in multiple large cohort studies [11,13].

Our data support these findings, demonstrating that patients treated with the Gastrografin protocol had a mean hospital stay of 2.2 days compared with 4.6 days in the nonprotocol group. This aligns with prior randomized controlled trials, including a study by Long et al., in which patients who underwent a Gastrografin challenge protocol experienced significantly shorter hospital stays and faster resolution of symptoms [11]. Additionally, the average number of CT scans during admission was significantly lower in the Gastrografin group (1.0 vs. 1.4), reflecting more streamlined decision-making and reduced reliance on imaging. This finding is particularly relevant in community settings, where access to advanced imaging may be delayed or limited.

Although our study showed only a nonsignificant reduction in surgical intervention and 30-day readmissions, the trend toward lower operative rates (4.2% vs. 21.2%) has clinical importance and warrants further investigation. This observation supports the notion that Gastrografin not only aids diagnosis but may also facilitate earlier resolution of partial obstructions, potentially avoiding surgery in some patients. Similar findings have been reported by Long et al. and Cohen et al., who demonstrated lower intervention rates and improved outcomes in patients managed with a Gastrografin protocol for adhesive SBO [9,11]. The relatively small sample size of this study likely limited the statistical power to detect differences in operative rates.

From a cost perspective, reducing LOS and CT imaging has substantial financial implications, improving bed turnover and hospital efficiency. Furthermore, decreased CT utilization reduces cumulative radiation exposure, aligning with ongoing efforts in radiation safety and stewardship.

Limitations

This study has several limitations. The use of a retrospective control group introduces potential biases related to changes in clinical practice over time. Although the same group of surgeons managed patients across both cohorts, unmeasured practice variations may still have influenced outcomes. Despite matching inclusion and exclusion criteria, unintended confounders could not be completely eliminated. Additionally, the relatively small protocol group (n = 24) limits generalizability. Larger, multicenter studies are needed to validate these findings and refine selection criteria for the Gastrografin challenge.

## Conclusions

This study demonstrates that the implementation of a standardized Gastrografin protocol for the management of adhesive SBO at a community hospital significantly reduces hospital LOS and CT scan utilization. The protocol provided a consistent and efficient framework for evaluating and managing patients, streamlining care without compromising safety. Although reductions in operative intervention and 30-day readmission did not reach statistical significance, the observed trends suggest potential clinical benefits worthy of further investigation. A multicenter randomized controlled trial with >80% power would be a logical next step to validate these findings.

These results underscore the value of adopting evidence-based pathways in resource-constrained settings to optimize patient outcomes and operational efficiency. By reducing reliance on repeat imaging and expediting clinical decision-making, standardized protocols such as the one described here offer a practical and scalable approach for improving SBO management. Future multicenter studies with larger sample sizes could further validate these findings and support broader implementation across community healthcare systems.
